# Elevated First-Trimester Total Bile Acid is Associated with the Risk of Subsequent Gestational Diabetes

**DOI:** 10.1038/srep34070

**Published:** 2016-09-26

**Authors:** Wolin Hou, Xiyan Meng, Weijing Zhao, Jiemin Pan, Junling Tang, Yajuan Huang, Minfang Tao, Fang Liu, Weiping Jia

**Affiliations:** 1 Shanghai Key Laboratory of Diabetes, Department of Endocrinology & Metabolism, Shanghai Jiao-Tong University Affiliated Sixth People’s Hospital, Shanghai Clinical Medical Center of Diabetes, Shanghai Key Clinical Center of Metabolic Diseases, Shanghai Institute for Diabetes, Shanghai, China; 2 Department of Obstetrics and Gynecology, Shanghai Clinical Center for Severe Maternal Rescue, Shanghai Jiao-Tong University Affiliated Sixth People’s Hospital, Shanghai, China

## Abstract

The aim of the current study is to assess whether total bile acid (TBA) level in first trimester pregnancy is associated with gestational diabetes mellitus (GDM). Biochemical parameters including serum TBA of 742 pregnant women were collected within 12 weeks of gestation and compared. At 24–28th weeks of gestation, 75 g oral glucose tolerance test (OGTT) was performed. The perinatal data of 330 women were collected. The results demonstrated women with GDM (n = 268) had higher first-trimester serum levels of TBA compared with healthy subjects (n = 474) (2.3 ± 1.4 μmol/L *vs.* 1.9 ± 1.0 μmol/L, *P* < 0.001). TBA was independently associated with GDM [adjusted odds ratio (AOR), 1.38; 95% confidence interval (CI), 1.18–1.61, *P* < 0.001]. Compared to the first category of TBA, women in the highest category had a marked increase in risk for GDM (AOR, 7.72; 95% CI, 3.22–18.50, *P* < 0.001). In conclusion, higher first-trimester TBA levels, even within normal range, may help indicate increased risk of GDM.

The incidence of gestational diabetes mellitus (GDM) is gradually increasing worldwide^1^, ranging from 3% to 14%. Furthermore, the occurrence in the cities of north China is as high as 9.3% in all pregnant women^2^. Hyperglycemia during pregnancy leads to latent harmful impact on mothers such as preeclampsia, increased caesarean rates, macrosomia, premature rupture of membrane (PROM) and the development of type 2 diabetes mellitus (T2DM)^3^. Strategies to prevent GDM have great potential to prevent or delay the onset of overt diabetes. Insulin resistance normally increases during pregnancy and resolves upon delivery^4^. GDM develops due to an insufficient adaptation to growing insulin requirements and often accompanies with obesity and other risk factors for adverse pregnancy outcomes^5^.

There were several proposed potential biomarkers reflecting increased hazard for GDM from previous researches. For instance, prepregnancy low adiponectin concentration, a marker of decreased insulin sensitivity and altered adipocyte endocrine function, may identify women at high risk for GDM^6^. Sridhar, S.B. *et al*. reported that γ-glutamyltransferase (γ-GT) measured before pregnancy may help to identify women at increased risk for subsequent GDM^7^. In a large prospective cohort study, researchers reported that elevated asalanine aminotransferase (ALT) levels in the first trimester can be used to identify high risk women for GDM^8^. Another large retrospective study showed that higher levels of fasting blood glucose in first trimester were associated with increased risk of GDM, cesarean section, and macrosomia^9^. A case-control study performed by Thadhani, R. *et al*. demonstrated maternal follistatin-like-3 (FSTL3) levels, an inhibitor of activin and myostatin involved in glucose homeostasis, are decreased in first trimester and associated with subsequent GDM^10^. Although treatment of GDM in the second and third trimester of pregnancy improves some adverse perinatal outcomes^11^,^12^, earlier detection of women at higher risk for GDM through biomarker measurement allows more time for diet, medication and excercise intervention.

Bile acids (BAs) include primary BAs and secondary BAs. Primary BAs including cholic acid (CA), chenodeoxycholic acid (CDCA) are produced from cholesterol in the liver. Most of them are conjugated with taurine and glycine, stored in the gallbladder, and secreted into the duodenum upon meal stimulation and digest lipids and lipid-soluble vitamins. Some primary BAs are transformed into secondary BAs including deoxycholic acid (DCA) and lithocholic acid by gut microbiome^13^. In recent years, BAs have been indicated as important regulatory molecules of whole-body metabolism, increasing energy expenditure and preventing obesity, insulin resistance, and T2DM by activating specific nuclear and cell surface receptors such as the farnesoid X receptor (FXR) and the G protein-coupled receptor (TGR5), respectively^14^,^15^. Brufau, G. *et al*. reported that T2DM subjects had greater CA synthesis rates and enlarged DCA pool size^16^. A recent study also found that BAs were nearly twofold elevated in T2DM patients compared with healthy subjects, and the disproportion in BAs was associated with insulin resistance^17^. Although total bile acid (TBA) has been known to correlate with features of insulin resistance and the development of T2DM, and insulin resistance is considered as the main mechanism underlying the pathophysiology of GDM, the change of TBA level during pregnancy and its relationship with the risk of GDM remain unclear. Thus, the aim of the present study was to clarify whether TBA at early pregnant stage is associated with the development of GDM.

## Results

### Subject characteristics

In this nested case-control study, a total of 742 pregnant women [mean age 30.1 ± 3.8 years, mean prepregnancy body mass index (BMI) 22.7 ± 3.3 kg/m^2^] were enrolled in this study. The clinical characteristics of control and GDM patients are shown in Table 1. In women who developed GDM, the incidence of diabetes family history and multiparity history before pregnancy were significantly higher. There were significant differences in age, prepregnancy BMI, systolic blood pressure (SBP), diastolic blood pressure (DBP), glycosylated hemoglobin A1c (HbA1c), γ-GT, cholinesterase (ChE), TBA, triglyceride (TG), fasting insulin, 1- and 2-hour insulin values, fasting plasma glucose, 1- and 2-hour glucose levels, homeostasis model assessment of insulin resistance index (HOMA-IR) and homeostasis model assessment of β cell insulin secretion (HOMA-β) (all *P* < 0.05). One hundred and seventy nine women with GDM and 151 women without were followed up until delivery, their perinatal data are shown in Table 2. There were significant differences in age, prepregnancy BMI, SBP, DBP, TBA, ChE, and TG between the two groups. The incidence of caesarean section and macrosomia and infant birth weight were significantly higher in women with GDM than healthy pregnant women (caesarean section, 26.4% *vs.* 17.3%*, P* = 0.045; macrosomia, 11.9% *vs.* 3.4%, *P* = 0.003; infant birth weight, 3385.3 ± 489.9 g *vs.* 3279.4 ± 417.7 g*, P* = 0.035). Other adverse pregnancy outcomes such as preeclampsia and PROM showed no marked difference.

### Binary logistic regression analysis of the association of TBA with clinical variables and adverse pregnancy outcomes

In order to find out which factors were independently associated with GDM and other adverse pregnancy outcomes, binary logistic regression was performed (Table 3). A significant independent association was found between serum TBA and GDM (OR, 1.31; 95% CI, 1.15–1.50, *P* = 0.008). Moreover, age, BMI, SBP, DBP, ChE and TG were found to be related to GDM. In the macrosomia subgroup, TBA (OR, 1.53; 95% CI, 1.13–2.08, *P* = 0.006) and BMI (OR, 1.25; 95% CI, 1.11–1.40, *P* < 0.001) were independent risk factors. In the caesarean subgroup, only BMI was identified as an independent risk factor (OR, 1.13; 95% CI, 1.04–1.23, *P* = 0.004). In the preeclampsia subgroup, age (OR, 1.21; 95% CI, 1.01–1.44, *P* = 0.036), DBP (OR, 1.10; 95% CI, 1.02–1.15, *P* = 0.016) and ChE (OR, 1.02; 95% CI, 1.00–1.03, *P* = 0.016) were independent risk factors. No significant correlation between those clinical variables and PROM was found. After adjustment for age, BMI, SBP, DBP, TG and other potential confounders, TBA was still an independent risk factor for GDM (OR, 1.38; 95% CI, 1.18–1.62, *P* < 0.001).

### The association between TBA and other clinical and biochemical characteristics at prenatal visit and delivery

A Spearman’s correlation analysis showed that serum TBA level was positively associated with HOMA-IR (r = 0.08), HOMA-β (r = 0.08) and neonatal weight (r = 0.12) (all *P* < 0.05). Multiple stepwise regression analysis showed that age (β = −0.075, *P* = 0.041), BMI (β = 0.099, *P* = 0.007), 1-hour glucose level (β = 0.110, *P* = 0.003) and 2-hour glucose level (β = 0.145, *P* < 0.001) were independent risk factors for TBA in all pregnant women.

### Receiver operating characteristic (ROC) analysis of clinical variables for GDM

ROC curve analysis revealed that area under curve (AUC) of serum TBA for indicating GDM was 0.569 (95% CI, 0.526–0.612; *P* = 0.002), AUC of age, prepregnancy BMI, SBP, DBP, ChE and TG to indicate GDM were 0.585 (95% CI, 0.542–0.628; *P* < 0.001), 0.546 (95% CI, 0.502–0.589; *P* = 0.039), 0.565 (95% CI, 0.522–0.608; *P* = 0.003), 0.566 (95% CI, 0.522–0.610; *P* = 0.003), 0.600 (95% CI, 0.558–0.643; *P* < 0.001) and 0.552 (95% CI, 0.509–0.595; *P* = 0.018).

### The occurrence and risk of GDM in different serum TBA categories

**Figure 1:**
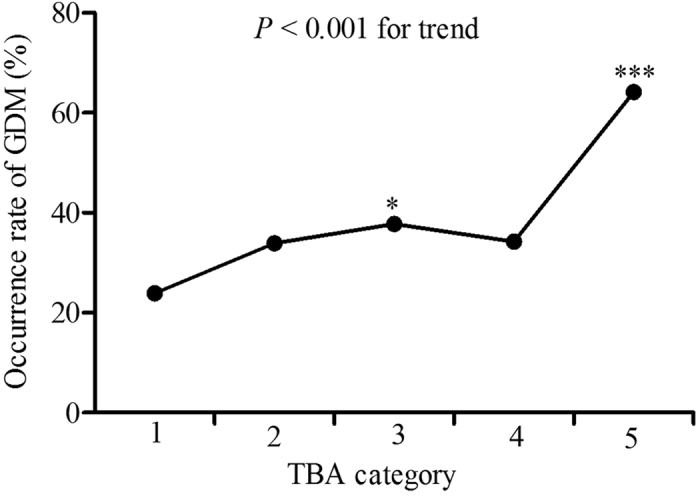
The occurrence of GDM in different first-trimester TBA categories. TBA categories are defined as follows: category 1 (<1.0 μmol/L, n = 71), category 2 (1.0–2.0 μmol/L, n = 354), category 3 (2.0–3.0 μmol/L, n = 188), category 4 (3.0–4.0 μmol/L, n = 76), category 5 (≥4.0 μmol/L, n = 53). *P < 0.05, ***P < 0.001 compared with the first TBA category. The overall *P* value was less than 0.001.

**Figure 2:**
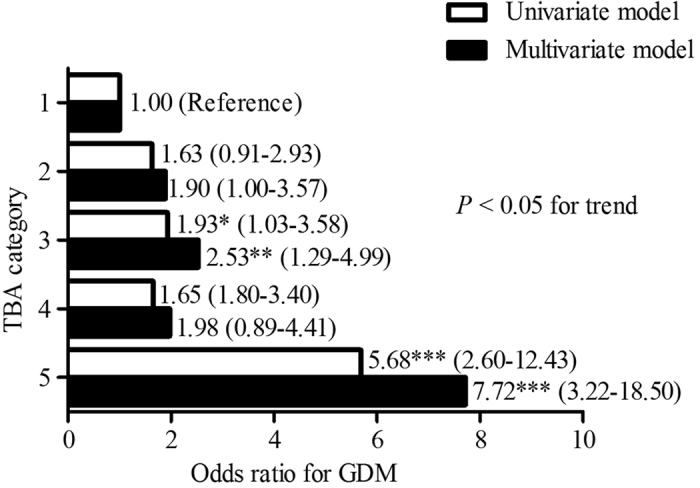
The risks for GDM in different first-trimester TBA categories. TBA categories are defined as follows: category 1 (<1.0 μmol/L, n = 71), category 2 (1.0–2.0 μmol/L, n = 354), category 3 (2.0–3.0 μmol/L, n = 188), category 4 (3.0–4.0 μmol/L, n = 76), category 5 (≥4.0 μmol/L, n = 53). *P < 0.05, **P < 0.01, ***P < 0.001 compared with the corresponding TBA category 1 (referent). The overall *P* value was less than 0.05.

TBA levels were analyzed in five categories: category 1 (<1.0 μmol/L, n = 71), category 2 (1.0–2.0 μmol/L, n = 354), category 3 (2.0–3.0 μmol/L, n = 188), category 4 (3.0–4.0 μmol/L, n = 76), category 5 (≥4.0 μmol/L, n = 53). The occurrence of GDM showed an increasing trend in the five categories (*P* < 0.001). Compared to the first category, the occurrence rates of GDM in the third and the fifth categories were significantly higher (37.8% *vs.* 23.9%, *P* = 0.024; 64.2% *vs.* 23.9%, *P* < 0.001, respectively) (Fig. 1). When considering TBA category 1 as the referent, women in the third and the fifth categories had markedly higher risk for GDM (OR, 1.93; 95% CI, 1.04–3.58, *P* = 0.038; OR, 5.68; 95% CI, 2.60–12.43, *P* < 0.001, respectively). After controlling for age, BMI and other confounders, the odds ratios for GDM were still significantly higher in category 3 (OR, 2.53; 95% CI, 1.29–4.99, *P* = 0.007) and category 5 (OR, 7.72; 95% CI, 3.22–18.50, *P* < 0.001) compared to category 1 (Fig. 2).

## Discussion

In the present study, we confirmed the link of TBA concentration with GDM in Chinese pregnant women. First-trimester TBA levels in women who developed GDM later were significantly higher than healthy women. Higher first-trimester TBA levels within normal range were independently related with increased risk of GDM, without a clear threshold. The highest category of TBA levels (TBA ≥ 4.0 μmol/L) reflected 6.72 increased risk for GDM compared to the lowest category (TBA < 1.0 μmol/L) after adjusting for age, BMI and other confounders.

A number of researches have investigated the relation between bile acid composition and level alteration in the progress of T2DM and its relationship with insulin resistance. Cariou, B. *et al*. reported 1.6-fold increases in DCA in individuals with T2DM and HOMA-IR was positively related with CDCA, CA and DCA after adjustment for other confounders^18^. Similarly, obese subjects with T2DM have higher synthesis of 12-hydroxy BAs (sum of CA, DCA, and their conjugates) compared to obese controls^19^. Our colleagues recently reported the levels of CDCA relative to total BA (CDCA%) were significantly higher in obese individuals with T2DM than non-T2DM obese subjects^20^. In the present research, BAs, which was known to be related to the development of T2DM, was also associated with HOMA-IR and GDM. It has been reported that BAs remained almost unchanged during the first trimester and were slightly increased, within the normal range, in the second and third trimester of pregnancy compared with non-pregnant women^21^. Due to the insusceptible nature of TBA to pregnancy, elevated first-trimester TBA level may reflect an underlying, more severe insulin resistance at early pregnancy stage in women who developed GDM.

Many of the beneficial effects of BAs on glucose metabolism are mediated via activating two receptors, FXR and TGR5. In *vivo* research showed FXR activation stimulates the insulin/Akt pathway in adipose tissue and skeletal muscle of mice, thus improve peripheral insulin sensitivity^22^. Other animal studies found that bile acid sequestrants colesevelam and resin stimulated glucagon-like peptide-1 (GLP-1) release through activation of TGR5, resulting in alleviative insulin resistance in diet-induced obese mice^23^,^24^. BA sequestrants, which block BA re-absorption in the gut and cause a compensatory increase in BA synthesis, are developed to treat hypercholesterolemia and improve glycemic status in T2DM^25^,^26^,^27^. These findings suggested that up-regulated TBA concentration might be protective at the early stage of GDM in order to ameliorate insulin resistance. On the other hand, the synthesis of BA was dependent on glucose through the cholesterol pathway. Glucose supplied the substrates including acetyl-coenzyme A and nicotinamide adenine dinucleotide phosphate (NADPH) and energy required for cholesterol synthesis^28^. In our study, postprandial glucose levels were independently correlated with TBA concentration. The increase in TBA might be a result of elevation of cholesterol production in liver following an increase in glucose supply. Besides, Gerhard, G.S. *et al*.^29^ hypothesized that an impairment in the enterohepatic circulation of BAs possibly present in diabetic state. Taken together, further studies are necessary to profoundly understand the underlying mechanism of TBA in the progress of GDM.

Maternal hyperglycemia less severe than overt diabetes is related to clinically important perinatal disorders such as macrosomia, primary cesarean delivery, premature delivery, neonatal hypoglycemia^30^. In the present study, women in GDM group had significantly higher incidence of caesarean section and macrosomia. The development of GDM could be predicted from maternal age and prepregnancy BMI^31^. In our study, age and prepregnancy BMI of the GDM group were higher than control group, and both of them were independent associated factors for GDM. Sridhar, S.B. *et al*. reported that pregravid γ-GT may help to identify women at increased risk for GDM^7^. Another research indicated that elevated ALT levels in the first trimester can be used to identify high risk women for subsequent GDM^8^. In the current study, γ-GT levels among women who developed GDM were similarly higher than non-GDM women, but it was not found to be an independent risk factor for GDM. This result suggested that γ-GT levels, at least determined during pregnancy, was not associated with GDM. And in our research, ALT levels in GDM group were slightly elevated, but the difference did not reach statistical significance. The inconsistence of our results with previous study may be due to relatively small sample size.

Although first-trimester HbA1c levels were measured for preliminary screening of overt diabetes, we did not consider HbA1c as predictor of GDM. Firstly, hemoglobin (Hb) concentration fluctuated during pregnancy. Blood volume increases during pregnancy and the gradual increase in blood volume begins in the first trimester, so first-trimester hemoglobin levels may be decreased because of pregnancy^32^. Thus, HbA1c may not truly reflect the glucose control during past 8–12 weeks in pregnant women. Secondly, HbA1c assay were not standardized in China. Thirdly, due to the disparities of HbA1c measuring methods and low quality control, HbA1c was not recommended as diagnostic criteria of diabetes according to the guideline of Chinese Diabetes Society (CDS), and was not suitable for diagnosing gestational diabetes mellitus. Thus, we did not focus on using HbA1c for early GDM detection.

There were some limitations of our study. Firstly, some other confounding factors of GDM such as diet and physical activity were not excluded. Secondly, we did not further test individual BAs and analyze their relationship with GDM. Moreover, serum TBA levels were measured only in a fasting state. Serum TBA can increase 4.5- to 6-fold during the first 30 minutes after a single oral glucose tolerance test^33^. The profile of postprandial TBA after a standard diet may be considered as a better risk factor for GDM. Thirdly, the ethnicity of the study population was relatively monotonous as we only recruited pregnant women from Shanghai. A well-designed, population-based prospective study should be conducted to overcome these limitations.

In summary, based on the results above, we can draw the conclusions that serum TBA level in first-trimester pregnancy was independently associated with the subsequent occurrence of GDM, elevated plasma TBA level, even within the normal reference range, identified women at high risk for GDM. Moreover, because of the feasibility, simplicity and convenience of its assay, serum TBA level can be a potentially new biomarker distinguishing women at high and low risk for GDM at early pregnancy.

## Methods

### Subject

It was a nested case-control study of subjects enrolled in a prospective cohort of pregnant women. In brief, from January 2013 to August 2015, we set up a cohort of pregnant women who received prenatal care at the Department of Obstetrics and Gynecology of Shanghai Jiao-Tong University Affiliated Sixth People’s Hospital. Their clinical and biochemical profiles longitudinally were documented from antenatal visit to delivery. To clarify the association between first-trimester TBA levels and GDM, we conducted a nested case-control study. Diagnostic 75-g, 3-hour oral glucose tolerance test (OGTT) was performed at 24–28th weeks of gestation. GDM was diagnosed when one or more of the following plasma glucose values were met or exceeded: fasting, 5.1mmol/L, 1 hour, 10.0 mmol/L, and 2 hours, 8.5 mmol/L, according to the criteria established by the International Association of Diabetes and Pregnancy Study Group (IADPSG)^34^. Women with alcohol consumption, preconceptional diabetes, intrahepatic cholestasis of pregnancy, chronic or serious acute infections, cardiovascular disease, hematological diseases, severely impaired liver or kidney function or if they have been tested for positive hepatitis C antibodies or HIV were excluded. The study was approved by the Ethics Committee of the Shanghai Jiao-Tong University Affiliated Sixth People’s Hospital. The informed consents were completed by all the participants. The methods were performed in accordance with the Declaration of Helsinki.

### Data collection

All the participants completed a questionnaire that collected general background information including present and previous illness, reproductive history, medication, alcohol consumption and smoking status. Height, weight and blood pressure were assessed on a standardized form by the same physician during the health check-up. BMI was calculated as BMI = body weight (in kg)/height (in m^2^). Macrosomia was defined as birth weight ≥4,000 g. Preeclampsia was defined according to new-onset hypertension and proteinuria, which appeared after 20 weeks of gestation. PROM was diagnosed when membranes ruptured before the onset of labor.

### Laboratory measurements

Blood samples were drawn after an overnight fast within 12 weeks of gestation. All biochemical parameters including TBA were analyzed in the same serum sample. HbA1c was determined by high-pressure liquid chromatography and glycated serum albumin (GA) was measured by the liquid enzymatic assay, the linearity ranges were 3.5–20% and 5–60%. Serum TBA concentration was determined by enzymatic method using an automatic analyzer (7600–020 biochemistry automatic analyzer, Hitachi, Tokyo, Japan), the linearity range was 0–180 μmol/L and the normal range was 0 to 10 μmol/L, the intra-assay and inter-assay coefficients of variation for TBA were less than 5% and 10%. Other biochemical indices evaluating hepatorenal function such ALT, aspartate aminotransferase (AST), γ-GT, ChE, blood urea nitrogen (BUN), creatinine (Cr), uric acid (UA) and serum lipids including TG, total cholesterol (TC), high-density lipoprotein cholesterol (HDL-C) and low density lipoprotein cholesterol (LDL-C) were performed by enzymatic method, the linearity ranges were 0–450 U/L, 0–450 U/L, 15–232 U/L, 0–11000 U/L, 0–43 mmol/L, 0–10000 μmol/L, 0–1500 μmol/L, 0–11.3 mmol/L, 0–20.68 mmol/L, 0–3.88 mmol/L, 0–12.9 mmol/L, respectively. Plasma glucose values were measured by glucose oxidase method, the linearity range was 0–35 mmol/L. Insulin concentrations were determined with a 2-site chemiluminescent enzyme immunometric assay for the immulite automated analyzer (Diagnostic Products, Los Angeles, CA), the linearity range was 0.02–1000 μU/ml. The intra-assay and inter-assay coefficients of variation for all biochemical indexes were less than 10%. The degree of insulin resistance was measured by HOMA-IR, calculated using the following formula: fasting glucose (mmol/l) × fasting insulin (μU/ml)/22.5. The HOMA-β was used for the assessment of insulin secretion, and it was calculated as [20 × fasting insulin (μU/mL)]/[fasting glucose (mmol/L)−3.5].

### Statistical analysis

Data were expressed as mean ± standard deviation (SD) for continuous variables and percentages (%) for categorical variables. Differences between groups were evaluated with Student’s t test or Mann-Whitney *U* test for continuous variables and Chi-square test or Fisher’s exact test for categorical variables. The association between TBA and other characteristics at prenatal visit and delivery was evaluated with Spearman correlation and partial correlation analysis. Binary logistic regression analysis was performed to evaluate the odds ratios (OR) and 95% confidence intervals (CIs) in univariable and multivariable analysis. Multiple stepwise regression analysis was used to investigate the influence of different variables on TBA. All the statistical analysis was performed by SPSS 21.0 (SPSS Inc., Chicago, IL). A two-sided *P* < 0.05 was considered statistically significant.

## Additional Information

**How to cite this article**: Hou, W. *et al*. Elevated First-Trimester Total Bile Acid is Associated with the Risk of Subsequent Gestational Diabetes. *Sci. Rep.* **6**, 34070; doi: 10.1038/srep34070 (2016).

## Figures and Tables

**Table 1:** Comparison of clinical characteristics and biochemical indexes of GDM and healthy pregnant women.

Group	Control	GDM	*P* value
N	474	268	
Age (years)^&^	29.8 ± 3.8	30.8 ± 3.8	0.001^†^
Prepregnancy BMI (kg/m^2^)^&^	22.5 ± 3.3	23.1 ± 3.5	0.020*
SBP (mm Hg)^&^	110.4 ± 12.6	113.2 ± 13.1	0.004^†^
DBP (mm Hg)^&^	66.4 ± 9.4	68.6 ± 10.4	0.003^†^
Family history of diabetes (%)^&^	0.4	2.2	0.029^#^
Nulliparous (%)^&^	77.8	72.0	0.046^#^
Abortion history (%)	31.2	37.3	0.054^#^
GA (%)	11.6 ± 1.4	11.7 ± 1.4	0.950^†^
HbA1c (mmol/mol)^&^	30.0 ± 3.1	32.0 ± 5.3	<0.001^†^
HbA1c (%)^&^	4.9 ± 0.3	5.1 ± 0.7	<0.001^†^
ALT (U/L)	17.7 ± 11.9	19.2 ± 15.0	0.158^†^
AST (U/L)	19.3 ± 6.9	19.4 ± 6.9	0.873^†^
γ-GT (U/L)^&^	15.2 ± 9.0	17.3 ± 11.2	0.006^†^
ChE (U/L)^&^	267.5 ± 49.8	285.4 ± 51.2	<0.001*
TBA (μmol/L)^&^	1.9 ± 1.0	2.3 ± 1.4	<0.001^†^
TBil (μmol/L)	8.2 ± 2.8	8.1 ± 2.9	0.480^†^
PAB (mg/L)	238.2 ± 42.2	243.4 ± 43.2	0.112^†^
BUN (mmol/L)	2.7 ± 0.7	2.6 ± 0.6	0.278^†^
Cr (μmol/L)	44.0 ± 6.0	43.2 ± 6.2	0.083^†^
UA (μmol/L)	203.8 ± 44.2	209.9 ± 44.6	0.070^†^
TC (mmol/L)	4.9 ± 0.9	4.9 ± 1.0	0.727^†^
TG (mmol/L)^&^	1.7 ± 1.0	1.9 ± 1.1	0.037^†^
HDL-C (mmol/L)	1.8 ± 0.4	1.8 ± 0.4	0.072^†^
LDL-C (mmol/L)	2.5 ± 0.7	2.5 ± 0.7	0.788^†^
75-gOGTT			
Gestational age at OGTT (weeks)	26.7 ± 1.4	26.7 ± 1.3	0.967^†^
Insulin 0 h (μU/ml)^&^	8.5 ± 4.2	11.1 ± 5.8	<0.001^†^
Insulin 1 h (μU/ml)^&^	70.9 ± 40.7	89.4 ± 54.0	<0.001^†^
Insulin 2 h (μU/ml)^&^	55.9 ± 37.1	91.1 ± 65.6	<0.001^†^
Glucose 0 h (mmol/L)^&^	4.5 ± 0.3	5.0 ± 0.5	<0.001^†^
Glucose 1 h (mmol/L)^&^	7.9 ± 1.2	9.8 ± 1.6	<0.001*
Glucose 2 h (mmol/L)^&^	6.3 ± 1.2	8.2 ± 1.6	<0.001*
HOMA-IR^&^	1.7 ± 0.9	2.5 ± 1.4	<0.001^†^
HOMA-β (%)^&^	1.9 ± 1.4	1.7 ± 1.1	0.035^†^

GDM, gestational diabetes mellitus; BMI, body mass index; SBP, systolic blood pressure; DBP, diastolic blood pressure; GA, glycated albumin; HbA1c, glycosylated hemoglobin; ALT, alanine aminotransferase; AST, aspartate aminotransferase; γ-GT, γ-glutamyltransferase; ChE, cholinesterase; TBA, total bile acid; TBil, total bilirubin; PAB, prealbumin; BUN, blood urea nitrogen; Cr, creatinine; UA, uric acid; TC, total cholesterol; TG, total triglycerides; HDL-C, high-density lipoprotein cholesterol; LDL-C, low-density lipoprotein cholesterol; HOMA-IR, homeostasis model assessment of insulin resistance index; HOMA-β, homeostasis model assessment of beta cell insulin secretion. Data represent means ± SD or percentage (%). *Derived from Student’s t- test. ^#^Derived from Chi-square test or Fisher’s Exact Test. ^†^Derived from Mann-Whitney U- test. ^&^Significant at *P* < 0.05 level.

**Table 2:** Comparison of pregnancy outcomes of GDM and healthy pregnant women.

Group	Control	GDM	*P* value
N	179	151	
Age (years)^&^	29.9 ± 3.7	31.2 ± 3.9	0.001^†^
Prepregnancy BMI (kg/m^2^)^&^	20.8 ± 2.9	22.5 ± 2.9	<0.001*
SBP (mm Hg)^&^	110.3 ± 12.6	113.9 ± 11.8	0.008*
DBP (mm Hg)^&^	66.8 ± 9.7	69.3 ± 10.5	0.022*
TBA (μmol/L)^&^	1.6 ± 0.8	2.2 ± 1.3	<0.001^†^
ChE (U/L)^&^	269.5 ± 47.3	290.5 ± 53.3	<0.001*
UA (μmol/L)	205.9 ± 43.6	210.2 ± 42.9	0.507^†^
TC (mmol/L)	4.8 ± 1.0	4.8 ± 0.9	0.986*
TG (mmol/L)	1.5 ± 1.2	1.7 ± 0.8	0.011^†^
Gestational age at delivery (weeks)	38.9 ± 1.3	39.0 ± 1.3	0.453^†^
Caesarean section (%)^&^	17.3	26.4	0.045^#^
PROM (%)	10	25.3	0.224^#^
Preeclampsia (%)	2.8	2.6	0.605^#^
Amount of postpartum hemorrhage (ml)	321.2 ± 117.2	326.0 ± 204.0	0.787^†^
AFI (cm)	12.1 ± 3.2	12.4 ± 3.4	0.435^†^
Offspring			
BPD (mm)	92.8 ± 4.1	92.5 ± 3.9	0.451^†^
Weight (g)^&^	3279.4 ± 417.7	3385.3 ± 489.9	0.035*
Macrosomia (%)^&^	3.4	11.9	0.003^#^
Apgar score	10 ± 0.1	9.9 ± 0.3	0.122^†^

GDM, gestational diabetes mellitus; BPD, biparietal diameter; PROM, premature rupture of membrane; AFI, amniotic fluid index. Data represent means ± SD or percentage (%). *Derived from Student’s t- test. ^#^Derived from Chi-square test or Fisher’s Exact Test. ^†^Derived from Mann-Whitney U- test. ^&^Significant at *P* < 0.05 level.

**Table 3:** Binary logistic regression analysis of the association of first-trimester TBA with clinical variables and adverse pregnancy outcomes.

	GDM	Macrosomia	Caesarean section	PROM	Preeclampsia
	OR (95% CI)	OR (95% CI)	OR (95% CI)	OR (95% CI)	OR (95% CI)
TBA	1.31 (1.15–1.50)**	1.53 (1.13–2.08)**	1.10 (0.87–1.39)	0.96 (0.70–1.32)	0.84 (0.42–1.69)
Age	1.07 (1.03–1.12)***	1.00 (0.90–1.11)	0.96 (0.90–1.03)	1.02 (0.93–1.11)	1.21 (1.01–1.44)*
BMI	1.06 (1.01–1.10)*	1.25 (1.11–1.40)***	1.13 (1.04–1.23)**	1.08 (0.96–1.21)	1.17 (0.97–1.40)
SBP	1.02 (1.01–1.03)**	1.02 (0.98–1.05)	1.02 (1.00–1.04)	1.01 (0.99–1.04)	1.03 (0.98–1.08)
DBP	1.02 (1.01–1.04)**	0.98 (0.94–1.02)	1.00 (0.98–1.03)	1.00 (0.96–1.03)	1.10 (1.02–1.15)*
ChE	1.01 (1.00–1.01)***	1.00 (1.00–1.01)	1.00 (0.99–1.00)	1.00 (1.00–1.01)	1.02 (1.00–1.03)*
TC	0.97 (0.83–1.14)	1.17 (0.77–1.77)	0.95 (0.71–1.26)	0.83 (0.59–1.18)	1.22 (0.64–2.34)
TG	1.17 (1.01–1.35)*	1.26 (0.96–1.64)	1.08 (0.85–1.35)	1.24 (0.89–1.72)	0.98 (0.50–1.93)
UA	1.00 (1.00–1.01)	1.00 (1.00–1.01)	1.01 (1.00–1.01)	1.00 (0.99–1.00)	1.01 (1.00–1.02)

GDM, gestational diabetes mellitus; BMI, body mass index; SBP, systolic blood pressure; DBP, diastolic blood pressure; ChE, cholinesterase; TBA,

total bile acid; UA, uric acid; TC, total cholesterol; TG, total triglycerides; PROM, premature rupture of membrane. *P < 0.05, **P < 0.01, ***P < 0.001.

## References

[b1] Metzger B. E. . International association of diabetes and pregnancy study groups recommendations on the diagnosis and classification of hyperglycemia in pregnancy. Diabetes care 33, 676–682 (2010). 20190296 10.2337/dc09-1848 PMC2827530

[b2] Chang Y., Chen X., Cui H., Zhang Z. & Cheng L. Follow-up of postpartum women with gestational diabetes mellitus (GDM). Diabetes Res Clin Pract 106, 236–240 (2014). 25271112 10.1016/j.diabres.2014.08.020

[b3] Metzger B. E. . Hyperglycemia and adverse pregnancy outcomes. N Engl J Med 358, 1991–2002 (2008). 18463375 10.1056/NEJMoa0707943

[b4] Ryan E. A., O’Sullivan M. J. & Skyler J. S. Insulin action during pregnancy. Studies with the euglycemic clamp technique. Diabetes 34, 380–389 (1985). 3882502 10.2337/diab.34.4.380

[b5] Buchanan T. A., Xiang A., Kjos S. L. & Watanabe R. What is gestational diabetes? Diabetes care 30 Suppl 2, S105–S111 (2007). 17596457 10.2337/dc07-s201

[b6] Hedderson M. M. . Low prepregnancy adiponectin concentrations are associated with a marked increase in risk for development of gestational diabetes mellitus. Diabetes care 36, 3930–3937 (2013). 23990523 10.2337/dc13-0389 PMC3836148

[b7] Sridhar S. B. . Pregravid liver enzyme levels and risk of gestational diabetes mellitus during a subsequent pregnancy. Diabetes care 37, 1878–1884 (2014). 24795397 10.2337/dc13-2229 PMC4067389

[b8] Leng J. . Plasma Levels of Alanine Aminotransferase in the First Trimester Identify High Risk Chinese Women for Gestational Diabetes. Sci Rep 6, 27291 (2016). 27264612 10.1038/srep27291 PMC4893691

[b9] Riskin-Mashiah S., Younes G., Damti A. & Auslender R. First-trimester fasting hyperglycemia and adverse pregnancy outcomes. Diabetes Care 32, 1639–1643 (2009). 19549728 10.2337/dc09-0688 PMC2732138

[b10] Thadhani R. . First-trimester follistatin-like-3 levels in pregnancies complicated by subsequent gestational diabetes mellitus. Diabetes Care 33, 664–669 (2010). 20007937 10.2337/dc09-1745 PMC2827528

[b11] Landon M. B. . A multicenter, randomized trial of treatment for mild gestational diabetes. N Engl J Med 361, 1339–1348 (2009). 19797280 10.1056/NEJMoa0902430 PMC2804874

[b12] Crowther C. A. . Effect of treatment of gestational diabetes mellitus on pregnancy outcomes. N EnglJ Med 352, 2477–2486 (2005). 15951574 10.1056/NEJMoa042973

[b13] Catoi A. F., Parvu A., Muresan A. & Busetto L. Metabolic Mechanisms in Obesity and Type 2 Diabetes: Insights from Bariatric/Metabolic Surgery. Obes Facts 8, 350–363 (2015). 26584027 10.1159/000441259 PMC5644813

[b14] Hylemon P. B. . Bile acids as regulatory molecules. J Lipid Res 50, 1509–1520 (2009). 19346331 10.1194/jlr.R900007-JLR200 PMC2724047

[b15] Staels B. & Fonseca V. A. Bile acids and metabolic regulation: mechanisms and clinical responses to bile acid sequestration. Diabetes care 32 Suppl 2, S237–S245 (2009). 19875558 10.2337/dc09-S355 PMC2811459

[b16] Brufau G. . Improved glycemic control with colesevelam treatment in patients with type 2 diabetes is not directly associated with changes in bile acid metabolism. Hepatology 52, 1455–1464 (2010). 20725912 10.1002/hep.23831

[b17] Haeusler R. A., Astiarraga B., Camastra S., Accili D. & Ferrannini E. Human insulin resistance is associated with increased plasma levels of 12alpha-hydroxylated bile acids. Diabetes 62, 4184–4191 (2013). 23884887 10.2337/db13-0639 PMC3837033

[b18] Cariou B. . Fasting plasma chenodeoxycholic acid and cholic acid concentrations are inversely correlated with insulin sensitivity in adults. Nutr Metab (Lond) 8, 48 (2011). 21736725 10.1186/1743-7075-8-48 PMC3143920

[b19] Ferrannini E. . Increased Bile Acid Synthesis and Deconjugation After Biliopancreatic Diversion. Diabetes 64, 3377–3385 (2015). 26015549 10.2337/db15-0214 PMC4587641

[b20] Yu H. . Chenodeoxycholic Acid as a Potential Prognostic Marker for Roux-en-Y Gastric Bypass in Chinese Obese Patients. J Clin Endocrinol Metab 100, 4222–4230 (2015). 26425885 10.1210/jc.2015-2884

[b21] Jamjute P., Ahmad A., Ghosh T. & Banfield P. Liver function test and pregnancy. J Matern Fetal Neonatal Med 22, 274–283 (2009). 19330714 10.1080/14767050802211929

[b22] Cariou B. . The farnesoid X receptor modulates adiposity and peripheral insulin sensitivity in mice. J Biol Chem 281, 11039–11049 (2006). 16446356 10.1074/jbc.M510258200

[b23] Potthoff M. J. . Colesevelam suppresses hepatic glycogenolysis by TGR5-mediated induction of GLP-1 action in DIO mice. Am J Physiol Gastrointest Liver Physiol 304, 371–380 (2013). 10.1152/ajpgi.00400.2012 PMC3566618 23257920

[b24] Harach T. . TGR5 potentiates GLP-1 secretion in response to anionic exchange resins. Sci Rep 2, 430 (2012). 22666533 10.1038/srep00430 PMC3362799

[b25] Handelsman Y. . Colesevelam hydrochloride to treat hypercholesterolemia and improve glycemia in prediabetes: a randomized, prospective study. Endocr Pract 16, 617–628 (2010). 20634176 10.4158/EP10129.OR

[b26] Fonseca V. A., Rosenstock J., Wang A. C., Truitt K. E. & Jones M. R. Colesevelam HCl improves glycemic control and reduces LDL cholesterol in patients with inadequately controlled type 2 diabetes on sulfonylurea-based therapy. Diabetes care 31, 1479–1484 (2008). 18458145 10.2337/dc08-0283 PMC2494667

[b27] Yamakawa T., Takano T., Utsunomiya H., Kadonosono K. & Okamura A. Effect of colestimide therapy for glycemic control in type 2 diabetes mellitus with hypercholesterolemia. Endocr J 54, 53–58 (2007). 17102570 10.1507/endocrj.k05-098

[b28] Hişmioğullari A. A., Bozdayi A. M. & Rahman K. Biliary lipid secretion. Turk J Gastroenterol 18, 65–70 (2007). 17602352

[b29] Gerhard G. S. . A role for fibroblast growth factor 19 and bile acids in diabetes remission after Roux-en-Y gastric bypass. Diabetes care 36, 1859–1864 (2013). 23801799 10.2337/dc12-2255 PMC3687273

[b30] Crowther C. A. . Effect of treatment of gestational diabeteson pregnancy outcomes. N Engl J Med 352, 2477–2486 (2005). 15951574 10.1056/NEJMoa042973

[b31] van Leeuwen M. . Estimating the risk of gestational diabetes mellitus: a clinical prediction model based on patient characteristics and medical history. Bjog 117, 69–75 (2010). 20002371 10.1111/j.1471-0528.2009.02425.x

[b32] Scott D. E. Anemia during pregnancy. Obstet Gynecol Ann 1, 219 (1972). 4591070

[b33] Zhao X. . Changes of the plasma metabolome during an oral glucose tolerance test: is there more than glucose to look at? Am J Physiol Endocrinol Metab 296, E384–E393 (2009). 19066319 10.1152/ajpendo.90748.2008

[b34] American Diabetes Association. Standards of medical care in diabetes–2013. Diabetes care 36 Suppl 1, S11–S66 (2013). 23264422 10.2337/dc13-S011 PMC3537269

